# Influence of the levonorgestrel-releasing intrauterine system on the risk of breast cancer: a systematic review

**DOI:** 10.1007/s00404-022-06640-y

**Published:** 2022-06-18

**Authors:** Aline Zürcher, Laura Knabben, Heidrun Janka, Petra Stute

**Affiliations:** 1 University of Bern, Bern, Switzerland; 2 Department of Obstetrics and Gynecology, Inselspital, University Clinic Bern, University of Bern, Friedbühlstrasse 19, 3010 Bern, Switzerland; 3 Medical Library, University Library Bern, University of Bern, Bern, Switzerland

**Keywords:** Breast cancer, Contraception, Hormone replacement therapy, Levonorgestrel, Levonorgestrel-releasing intrauterine system, Premenopause, Postmenopause

## Abstract

**Purpose:**

The intention of this systematic review was to analyze the literature on breast cancer (BC) and the use of the levonorgestrel-releasing intrauterine system (LNG-IUS).

**Methods:**

The literature was searched in Medline, Embase, Cochrane Library, CINAHL, Web of Science and ClinicalTrials.com and included search terms related to breast cancer and LNG-IUS. After elimination of duplicates, 326 studies could be identified and were assessed according to inclusion and exclusion criteria. In the end, 10 studies met the defined criteria and were included in the systematic review.

**Results:**

6 out of the 10 selected studies were cohort studies, three were case–control studies and one a systematic review/meta-analysis. 6 found a positive association between BC and the use of LNG-IUS. One study only found an increased risk for invasive BC in the subgroup of women aged 40–45 years. In contrast, three studies showed no indication of a higher BC risk.

**Conclusion:**

The results imply an increased BC risk in LNG-IUS users, especially in postmenopausal women and with longer duration of use. Positive effects of the LNG-IUS such as reduced risks for other hormonal cancers have been observed, were, however, not focus of this systematic review. The heterogeneity of the analyzed studies and vast number of confounding factors call for further investigations in this issue. Patients should be advised according to their individual risk profile and hormone-free alternatives may be considered for women with a history of BC.

## Introduction

With 2.3 million new cases per year worldwide, BC is the most common cancer among women [1]. Fortunately, the mortality rate decreased due to implementation of screening and improved therapies during the last decades. Multiple risk factors are known including age, genetic factors, breast density, number of births, alcohol consumption, as well as endogenous and exogenous hormone exposure e.g. in the context of hormone replacement therapy (HRT) or contraception [2]. Regarding HRT, there is already a considerable amount of information available: long-term WHI data showed a significantly decreased BC risk with oestrogen-only HRT. Meanwhile, women who took combined oestrogen and progestogen HRT had an increased risk of being diagnosed with BC [3]. While the risk of HRT and also combined oral contraceptives is relatively well understood, the effect of progesterone alone on the female breast remains controversial.

LNG-IUS is a contraceptive method based on progesterone but is also used for treatment of menorrhagia or endometrial protection in postmenopausal women undergoing oestrogen therapy. It has been shown that progestins combined with oestrogens substantially increase BC risk [4]. Even though the amount of levonorgestrel released by LNG-IUS is low and seems to achieve the lowest systemic progestin levels of all progestin-only methods and most common contraceptives [5, 6], side effects after insertion of LNG-IUS include amongst others breast tenderness [6]. A possible influence on the breast and therefore on the BC risk cannot yet be ruled out with certainty.

Since there are several studies with inconsistent results addressing the possible influence of LNG-IUS on BC, the objective of this systematic review was to collect and analyze the existing literature concerning this matter.

## Methods

To evaluate the association between the use of LNG-IUS and BC risk, a systematic review was performed. The screening and exclusion process was based on the PRISMA 2020 guidelines. The literature was peer-reviewed using the exclusion criteria.

All studies associated with BC risk and the use of LNG-IUS were included. Only articles in English, German or French were revised.

Duplicates as well as papers and reports without original data were considered ineligible and were excluded from the search. Data from patients known to be at high risk for developing BC were also not considered.

To identify all potentially relevant documents on the topic, systematic literature searches were designed and executed for the following information sources: besides the standard medical bibliographic databases Medline, Embase and the Cochrane Library, CINAHL and one interdisciplinary database, Web of Science, were searched. In addition, ClinicalTrials.gov, a database of clinical trials, was checked for unpublished trials on the topic. All searches were run on February 24th, 2021.

Search terms were identified by looking at subject headings, titles, abstracts and author keywords from a list of core references. An initial search strategy in Medline was drafted by a medical information specialist and tested against these core references to see if they were included in the search results. After refinement and consultation, search strategies were set up for each information source based on database-specific controlled vocabulary (thesaurus terms/subject headings) and textwords. No limits have been applied in any database considering study types, languages, publication years or any other formal criteria. Animal studies have been excluded.

The search concepts included were 1. “breast cancer” (in pre-, peri- and post-menopausal women) and 2. “levonorgestrel-releasing intrauterine systems”. Synonyms, acronyms and similar terms were used for all concepts in the textword search, as well as the thesaurus terms.

The final detailed search strategies are presented in the “Appendix”.

**Fig. 1:**
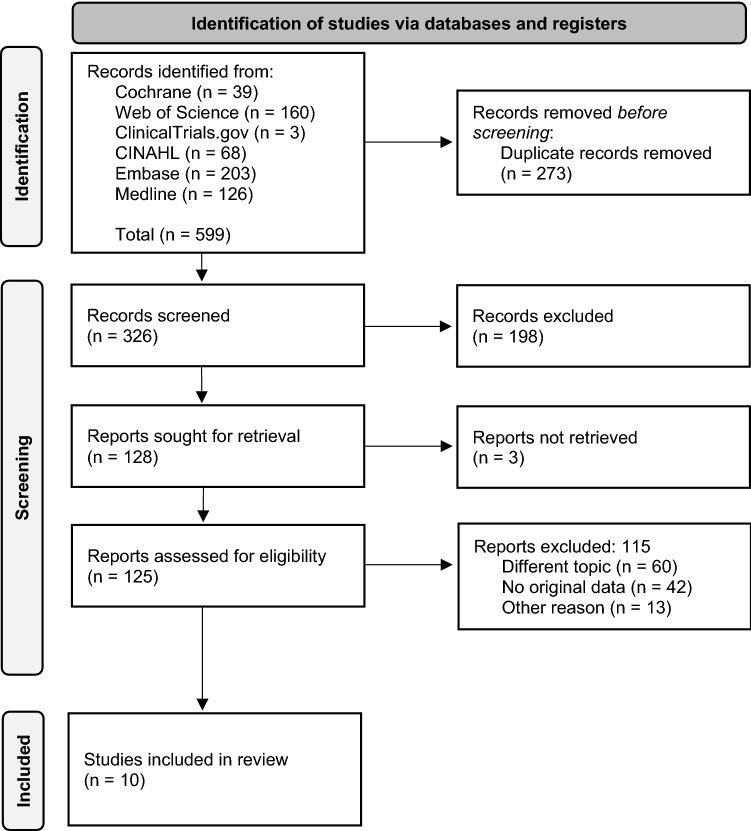
PRISMA flow chart

All identified citations were imported into EndNote and duplicates were removed. Subsequently, the screening of titles and abstracts as well as the eligibility assessment were performed. In the first screening process, all 326 studies were screened by title and abstract. 198 studies could thus already be excluded. In a second round, the remaining studies were tested against the inclusion criteria and assessed for eligibility. In the end, 10 studies were according to the four-eyes principle, considered eligible and therefore used for this systematic review (Fig. 1).

## Results

Overall, 326 studies were identified. Out of these, 10 studies [7–16] met the inclusion criteria and were thus selected (Table 1).

**Table 1:** Included studies

Author	Study design	Objective	Methods	Study duration /duration of LNG-IUS use	Study cohort	Levonorgestrel dosage and treatment regimen	Results	Summary
Conz, 2020 [7]	Systematic review and meta-analysis	Comparison of the literature on the use of LNG-IUS and the associated BC risk	The Downs and Black instrument was used for assessing the study quality and a random-effects model was performed for the meta-analysis (due to high study heterogeneity)	Various studies with different time of follow up and duration of LNG-IUS use	8 studies are included in the systematic review, 7 studies in the meta-analysis	52 mg LNG-IUS (20 μg/day) **Indication: variable**	Age < 50 years, OR = 1.12; 95% CI 1.02–1.22, *I*^2^ = 66%, *p* = 0.02	Increased risk for women < 50 years and > 50 years. The effect seems to be larger in older users
							Age ≥ 50 years, OR = 1.52; 95% CI 1.34–1.72, *I*^2^ = 0%, *p* = 0.84	
							For all women, the meta-analysis indicated an increased BC risk in LNG-IUS users, OR = 1.16; 95% CI 1.06–1.28, *I*^2^ = 78%, *p* < 0.01	
							**The calculated OR is not adjusted for the use of HRT**	
Siegelmann-Danieli, 2018 [8]	Retrospective cohort study	Perimenopausal women were assessed for LNG-IUS use and occurence of BC	5-year KM estimates	Time of follow up was 6.5 years in LNG-IUS users (starting at time of insertion) and 9.6 years in controls (starting from the day they reached the age of the matching case)	All Maccabi Healthcare Services female members aged 40–50 were included. LNG-IUS users were age-matched with two non-users. In total, these were 40.678 women (13.354 LNG-IUS users / 27.324 controls). Mean age in LNG-IUS users was 44.1 ± 2.6 and in controls 44.9 ± 2.8 years	52 mg LNG-IUS (20 μg/day)	Within 5 years after study entry, 136 (1.0%) BC cases occured in LNG-IUS users and 283 (1.0%) in controls. These included 16 DCIS and 120 invasive tumours in LNG-IUS users and in the controls 42 DCIS and 241 invasive tumours. For overall BC risk in LNG-IUS users and controls, 5-year KM estimates were 1.2% (SE 0.1%) and 1.1% (SE 0.06%), respectively (*p* = 0.23). The respective values for DCIS risk were 0.14% (SE 0.03%) and 0.16% (SE 0.03%), (*p* = 0.22). For invasive BC, the values were 1.06% (SE 0.1%) in LNG-IUS users vs. 0.93% (SE 0.06%) in controls (*p* = 0.051)	LNG-IUS does not increase the risk of BC overall or the risk of DCIS. LNG-IUS use was associated with a slightly increased risk of invasive BC tumours in the subgroup of younger women (40–45 years), while no significant effect was observed in those aged 46–50 years
						Women with prior, current or subsequent use of exogenous reproductive hormones (OC, HRT, fertility drugs, prophylactic use of tamoxifen) were excluded		
						**Indication: not clearly stated, but an estimated 90% used LNG-IUS as contraception. The remaining part was probably mainly treated for menorrhagia**	**The estimates exclude women with prior or subsequent exposure to female hormones, HRT included**	
Jareid, 2018 [9]	Prospective cohort study	Assessment of the risks of ovarian and endometrial cancer as well as BC in ever users and never users of LNG-IUS	RR with 95% CI estimated with Poisson regression	Mean time of follow up was 12.5 years. Mean duration of LNG-IUS use was 4 years but ranged from < 1 year to 14 years	104.318 women (9.144 ever users of LNG-IUS and 95.174 never users). Median age was 52 years	52 mg LNG-IUS (20 μg/day)	RR for BC = 1.03; 95% CI 0.91–1.17	Ever users of LNG-IUS had a strongly reduced risk of ovarian and endometrial cancer compared to never users, with no increased risk of BC
						The authors adjusted to risk factors such as ever use of other hormonal contraception or menopausal status	**RR is not adjusted for the use of HRT**	
						**Indication: not clearly stated, but probably contraceptive as well as medical reasons**		
Mørch, 2017 [10]	Prospective cohort study	Association between the use of hormonal contraception and the risk of invasive BC	RR with 95% CI estimated with Poisson regression plus bias analysis	Mean time of follow up was 10.9 ± 5.8 years Duration of LNG-IUS use ranged from less than 1 year to more than 10 years	1.797.932 women in Denmark between 15 and 49 years of age	52 mg LNG-IUS (20 μg/day)	RR for BC = 1.21; 95% CI 1.11–1.33	Overall, the risk of BC was higher among women who currently or recently used hormonal contraception. This includes the use of the LNG-IUS. The risk increased with longer durations of use. Nonetheless, absolute increases in risk were small
						Adjustment for several risk factors		
						**Indication: contraception**	**The calculated RR is not adjusted for the use of HRT. However, the participants are premenopausal and the use of HRT should not yet play an important role**	
Soini, 2016 [11]	Retrospective cohort study	Testing of the hypothesis that risk for lobular BC is elevated among LNG-IUS users	95% CI for the SIR were based on the assumption that the number of observed cases represents a Poisson distribution. A SIR with p < 0.05 was considered statistically significant	Mean time of follow up was 11 years. No exact information about actual duration of LNG-IUS use	93.843 Finnish women aged 30–49 years using LNG-IUS for treatment of menorrhagia (2.015 BC cases, of which 1.598 cases were of the invasive ductal histological type, 376 were invasive lobular cancers, and 41 were other histological types)	52 mg LNG-IUS (20 μg/day)	For ductal BC, SIR = 1.20; 95% CI 1.14–1.25 and for lobular BC SIR = 1.33; 95% CI 1.20–1.46. After two or more purchases of the LNG-IUS, the SIR for invasive lobular BC was 1.73 (81 cases; 95% CI 1.37–2.15, *p* < 0.001) and for invasive ductal cancer also 1.37 (286 cases; 95% CI 1.21–1.53, *p* < 0.001)	The results imply an excess risk of lobular but also ductal BC. The SIRs were higher in the subgroup of the LNG-IUS users who purchased the LNG-IUS at least twice
						All women included in this study were treated for menorrhagia. No adjustment for confounding factors such as family history of BC or use of exogenous hormones		
						**Indication: treatment for menorrhagia**	**The calculated SIR are not adjusted for the use of exogenous hormones**	
Heikkinen, 2015 [12]	Retrospective case–control study	Estimation of the association between use of exogenous hormones and BC risk	Conditional logistic regression was used to estimate OR and 95% CI	Mean time of follow up was 8 years (from 1 January 2000 to 31 December 2007)	25.560 Finnish women aged 22–60 years (5.927 BC cases / 19.633 controls). Median age was 57.5 years	52 mg LNG-IUS (20 μg/day)	OR for BC = 1.48; 95% CI 1.10–1.99	The study found positive associations with BC risk and exclusive use of LNG-IUS in postmenopausal women
						The authors adjusted for several risk factors		
						**Indication: not clearly stated**	**The calculated OR refers to the exclusive use of LNG-IUS**	
Soini, 2014 [13]	Retrospective cohort study	Association between premenopausal use of the LNG-IUS and cancer incidence with a special focus on endometrial adenocarcinoma	Standardized incidence ratio with 95% CI and Poisson regression	Mean time of follow up was 11 years. No exact information about actual duration of LNG-IUS use	93.843 Finnish women aged 30–49 years	52 mg LNG-IUS (20 μg/day)	SIR = 1.19; 95% CI 1.13–1.25 for BC among all LNG-IUS users and SIR = 1.40; 95% CI 1.24–1.57 among users with two LNG-IUS purchases	LNG-IUS is associated with a lower incidence of endometrial, ovarian, pancreatic, and lung cancer than expected. LNG-IUS use was associated with a higher incidence of BC, especially in the age categories of 45–54 years
						No adjustment for confounding factors such as family history of BC or use of exogenous hormones		
						**Indication: treatment for menorrhagia**	**The calculated SIR is not adjusted for the use of exogenous hormones**	
Dinger, 2011 [14]	Matched case–control study	Comparison of BC risk in LNG-IUS users and Cu-IUS users. The BC risk estimate was also compared with the risk of non-use of contraceptive methods, other progestin-only methods, and different hormonal contraceptives	Non-inferiority design, the null hypothesis to be tested was OR ≥ 1.5	Study duration was 8 years (January 2000 to December 2007) The duration of LNG-IUS use is not specified	25.565 German and Finnish women aged < 50 years (5.113 BC cases / 20.452 controls). Mean age of cases was 44.5 years and of controls 44.2 years	52 mg LNG-IUS (20 μg/day)	Crude and adjusted OR for the risk of BC among ever LNG-IUS users compared with ever Cu-IUS users were 1.04; 95% CI 0.93–1.17 and 0.99; 95% CI 0.88–1.12. Among current users (women who were using LNG-IUS at the time of diagnosis), crude and adjusted OR were 0.90; 95% CI 0.58–1.41 and 0.85; 95% CI 0.52–1.39	BC risk of LNG-IUS compared with Cu-IUS showed no indication of tumour promotion or tumour induction
						Adjustment for risk factors, including use of exogenous hormones		
						**Indication: not clearly stated. Probably primary use as contraceptive method, as short-term users and women who used LNG-IUS against endometrial hyperplasia were excluded in subanalyses**	**The calculated OR should not include women with HRT, as the participants were younger than 50 years**	
Lyytinen, 2010 [15]	Matched retrospective case–control study	Association between postmenopausal HRT and the risk for BC	OR with 95% CI calculated with conditional logistic regression analysis	Study duration was 12 years. LNG-IUS use ranged from < 3 to ≥ 5 years	39.824 Finnish women between 50–62 years (9.956 women diagnosed with first invasive BC/29.868 born at the same time but free of BC)	52 mg LNG-IUS (20 μg/day)	OR = 1.45; 95% CI 1.97–1.77. When the LNG-IUS was used as a complement to oestradiol, OR = 2.15; 95% CI 1.72–2.68	The association between HRT use and the risk for BC shows a large variation between various forms of HRT, the use of LNG-IUS alone may also carry a risk
						Additionally, many women used LNG-IUS as part of HRT		
						**Indication: postmenopausal HRT**	**The calculated OR refers to women with HRT but also LNG-IUS use alone and is adjusted for parity, age at the first birth and health care district**	
Backman, 2005 [16]	Cohort study (Re-analysis of post-marketing study)	Relationship between BC and use of LNG-IUS	95% CI based on the chi-square distribution and Fisher exact test	Study duration and time of LNG-IUS use was up to 10 years	17.360 Finnish women between 30 and 54 years of age, mean age was 35.4 years	52 mg LNG-IUS (20 μg/day)	The incidence rate per 100.000 woman-years was for the age groups 30–34 years 27.2 and 25.5, for 35–39 years 74.0 and 49.2, for 40–44 years 120.3 and 122.4, for 45–49 years 203.6 and 232.5, and for 50–54 years 258.5 and 272.6, in the levonorgestrel system group and in average Finnish female population, respectively	The study shows no indication of a higher BC risk between LNG-IUS users and average Finnish female population in any of the 5-year age groups
						No adjustment for risk factors, including the use of exogenous hormones		
						**Indication: not clearly stated**	**It is unclear whether the data were generated with or without HRT**	

The other studies were excluded due to e.g. focusing on contraception for women with a history of BC or discussing HRT for BC patients.

Six of the selected studies were cohort studies [8–11, 13, 16], three were case–control studies [12, 14, 15] and one a systematic review/meta-analysis [7]. Only one study was retrieved from Embase/Ovid [12], the rest was found in the database Medline [7–11, 13–16].

Most of the studies were performed in Finland [11–13, 15, 16]. One additional case–control study contained data from Finland as well as from Germany [14]. One cohort study stemmed from Norway [9], one from Denmark [10] and one from Israel [8]. The systematic review/meta-analysis was conducted in Brazil [7].

Primary endpoints of all studies were the occurrence of BC [7, 8, 10–12, 14–16], except for two studies where they further focused on other cancer risks such as ovarian and endometrial [9] as well as other types of cancer [13].

The sample size ranged from 17.360 [16] to 1.797.932 [10] women, mean time of follow up was 6.5 years [8] up to 12.5 years [9]. The studies assessed both pre- and postmenopausal women, as the youngest participants were only 15 years old at the time of study entry [10], the oldest 76 years [9]. Six out of the ten selected studies focused more on pre- or perimenopausal women [8, 10, 11, 13, 14, 16], two more on postmenopausal women [9, 15] and two included both age categories [7, 12].

The data on LNG-IUS use and BC incidence was obtained through self-administered questionnaires [9, 12, 14, 16], medical records/nationwide registries [8–15] or study databases [7].

Six studies found a positive association between the use of LNG-IUS and BC [7, 10–13, 15]. One study only stated a slightly increased risk for invasive BC in the subgroup of women aged 40–45 years [8]. Three studies observed no correlation between LNG-IUS and BC [9, 14, 16].

### BC risk in pre-/perimenopausal women using LNG-IUS

Three out of the six studies focusing on pre- or perimenopausal women found a positive association with LNG-IUS and BC [10, 11, 13]. One only found an elevated risk for invasive BC in women between 40 and 45 years [8]. Two studies found no positive association [14, 16].

A retrospective cohort study found an increased BC risk in premenopausal LNG-IUS users with standardized incidence ratio (SIR) = 1.19; 95% confidence interval (CI): 1.13–1.25 after a 10-year-follow up. After the second purchase of an LNG-IUS, the risk increased to SIR = 1.40; 95% CI 1.24–1.57. The higher prevalence of BC in premenopausal women using LNG-IUS occurred especially in the age categories from 45 to 54 years. It should be emphasized that the authors could not adjust for potential confounding factors—most importantly for the use of other exogenous hormones, e.g. HRT. Furthermore, these numbers were only collected from premenopausal women treated for menorrhagia, which may represent a selection bias [13].

Using a more heterogenous and larger study cohort involving all women in Denmark between 15 and 49 years old, a prospective cohort study also found positive associations with LNG-IUS use (and in general the use of hormonal contraception) and BC risk. The risk increased with longer duration of use, relative risk (RR) = 1.21; 95% CI 1.11–1.33. Additional adjustments for several risk factors did not change the estimates, which supports the results from the above-mentioned study [10].

Likewise a retrospective cohort study showed an increased risk for lobular BC with SIR = 1.33; 95% CI 1.20–1.46. After two or more purchases of the LNG-IUS, the SIR for invasive lobular BC was 1.73; 95% CI 1.37–2.15. The risk for invasive ductal BC was also elevated, with SIR = 1.20; 95% CI 1.14–1.25 and SIR = 1.37; 95% CI 1.21–1.53, respectively [11].

An Israeli cohort study supports the so far listed results: observing perimenopausal women only, the authors also found a slightly increased risk for invasive BC in the subgroup of women aged 40–45 years. The 5-year Kaplan–Meier (KM) estimates were 1.06% (standard error (SE) 0.1%) in LNG-IUS users vs. 0.93% (SE 0.06%) in controls, *p* = 0.051. However, no significant effect was observed in the age group 46–50 years and the study also showed no increased risk for ductal carcinoma in situ ? (DCIS) in their 5-year KM estimates [8].

Two studies observed no significant association at all between LNG-IUS use and BC in premenopausal women [14, 16]. There was no higher incidence of BC in LNG-IUS users compared to the average population in Finland [16] and compared to copper-containing Intrauterine System (Cu-IUS) users in Finland and Germany [14]. In comparison to the average female population, the incidence per 100,000 women years in LNG-IUS users was 27.2 and 25.5 for women aged 30–34 years. In the age group 35–39 years it was 74.0 and 49.2, in the group aged 40–44 years it was 120.3 and 122.4. Finally, BC incidence in the age group 45–49 years was 203.6 versus 232.5 and in the group for women aged 50–54 years, it was 258.5 and 272.6 [16].

The same conclusion was yielded when comparing women using LNG-IUS and Cu-IUS at the time of BC diagnosis: Odds ratio (OR) = 0.99; 95% CI 0.88–1.12. Among BC patients who were using LNG-IUS, the respective OR was 0.85; 95% CI 0.52–1.39. The authors adjusted for numerous risk factors such as family history of BC, age at menarche, HRT or oral contraceptive (OC) use. Comparing frequency of in situ or invasive ductal or lobular BC as well as tumour size and metastasis status, the case–control study with 25,565 German and Finnish women using either LNG-IUS or Cu-IUS found no difference [14].

Overall, the majority of the studies looking at BC risk in pre- or perimenopausal women found an increased incidence in LNG-IUS users, although not all of them considered other possible confounders. The risk seemed to increase with the purchase of more than one LNG-IUS. Nonetheless, the risk increase in each case was minimal.

### BC risk in postmenopausal women using LNG-IUS

Four studies were focusing primarily on postmenopausal women, although partly also including younger participants [7, 9, 12, 15]. Three studies found an increased BC risk in postmenopausal women [7, 12, 15], one additional study could not support this finding [9].

A population-based survey that estimated the association of exogenous hormones and BC risk stated a positive association between BC and use of LNG-IUS. The study found an elevated BC risk during exclusive use of LNG-IUS in postmenopausal women (OR = 1.48; 95% CI 1.10–1.99). As other possible risk factors like family history of BC, age at menarche, smoking, alcohol use and body mass index (BMI) were also considered, but the result could—according to the authors—not entirely be created by confounders [12].

This finding is supported by a retrospective case–control study, where the use of LNG-IUS alone as well as in complement to oestradiol caused a risk for BC (OR = 1.45; 95% CI 1.97–1.77 and OR = 2.15; 95% CI 1.72–2.68, respectively). Similar to the population-based survey, the authors also considered some confounders like parity, age at first birth and health care district [15]. Both results stemmed from women already diagnosed with BC [12, 15].

Finding no increased risk (RR = 1.03; 95% CI 0.91–1.17) was a prospective cohort study that assessed the risks of ovarian, endometrial and BC in ever users and never users of LNG-IUS [9].

Finally, a systematic review/meta-analysis pointed in turn towards a higher BC risk among LNG-IUS users aged 50 years or older. The authors compared eight studies with different study cohorts (all also mentioned in this review [8–12, 14–16]). Their meta-analysis indicated an increased BC risk in LNG-IUS users: for all women, OR = 1.16; 95% CI 1.06–1.28, *I*^2^ = 78%, *p* < 0.01. For women aged less than 50 years, OR = 1.12; 95% CI 1.02–1.22, *I*^2^ = 66%, *p* = 0.02 and for women aged more than 50 years, OR = 1.52; 95% CI 1.34–1.72, *I*^2^ = 0%, *p* = 0.84. However, the results should be interpreted with caution as some of the included studies showed methodological issues [7].

In conclusion, the BC risk in postmenopausal women using LNG-IUS is probably also increased, as three out of four studies focusing on this age group stated positive associations. Major confounding factors were also considered in the evaluations—nonetheless, the influence of HRT on the results is unclear.

### Risk for other cancers

When looking at other hormone-related cancers, ever users of LNG-IUS had a strongly reduced risk of ovarian and endometrial cancer compared to never users. For any hormone-related cancer, the RR in ever users was 0.86; 95% CI 0.77–0.97 [9]. Another cohort study also yielded a lower incidence of endometrial, ovarian, pancreatic and lung cancer in premenopausal women. However, they detected overall 188 excess cancer cases (more observed than expected) in LNG-IUS users during follow-up. Women with two LNG-IUS purchases had a 20% excess (76 cancer cases more than in non-users) [13].

### Quality of the included studies

The two prospective cohort studies showed different results: the first one stated no elevated risk for BC [9], the other one found an increased risk for LNG-IUS users [10]. The four retrospective cohort studies supported this finding [8, 11–13], although one of them only for the subgroup of women aged 40–45 years [8]. The systematic review/meta-analysis yielded similar results [7]. The two case–control studies were inconsistent with each other: the first one showed an increased BC risk for LNG-IUS users [15], the second, however, did not [14]. Likewise stating no positive association between LNG-IUS and BC was the re-analysis of a post-marketing study [16].

The seven studies stating a (partly) positive association with LNG-IUS and BC included a higher number of patients, ranging from 25,560 [12] up to almost 1.8 million women [10]. Only two of them did not adjust for the use of exogenous hormones such as HRT, both were using the same study cohort consisting of women treated for menorrhagia [11, 13]. The three studies which found no elevated BC risk in LNG-IUS users all included a relatively small number of cases, from 17,360 [16] to 104,318 [9]. Two of these studies did not adjust for known risk factors such as exogenous hormone exposure [9, 16].

In conclusion, the studies finding an increased BC risk in LNG-IUS users were mostly retrospective cohort studies with a tendency towards larger cohorts and less confounding factors such as HRT.

## Discussion

This systematic review showed that (1) the use of LNG-IUS seemed to increase BC risk, (2) with ORs up to 1.52; 95% CI 1.34–1.72 [7], this elevated risk was more evident in postmenopausal women and with longer durations of use and (3) confounding factors could have influenced the results.

Six of the ten studies included in this review stated a positive association between LNG-IUS and BC, although the risk increase was small. However, the included studies were very heterogenous. As each looked at various study cohorts with women at a different age and using LNG-IUS for different reasons (e.g. contraception, treatment for menorrhagia, HRT during menopause), it was difficult to compare them directly and draw clear conclusions. It would be necessary to verify the results in further studies, taking possible confounders into account. An extensive comparison to other hormonal contraceptive methods and their respective BC risk would also be interesting.

The BC risk increase becomes more evident in postmenopausal women and ranged up to OR = 1.52; 95% CI 1.34–1.72, *I*^2^ = 0%, *p* = 0.84 [7]. In combination with oestradiol within the framework of postmenopausal HRT it was even increased to OR = 2.15; 95% CI 1.72–2.68 [15]. The LNG-IUS could have influenced this finding, but it could also be distorted by other risk factors which become more numerous with higher age and thus increase the risk for BC in postmenopausal women.

Longer durations of use also seem to be a risk factor. After two or more purchases of an LNG-IUS, the SIR was up to 1.73; 95% CI 1.37–2.15, *p* < 0.001 [11].

Nonetheless, the selected studies were probably susceptible to confounding factors. Since LNG-IUS is used as part of HRT, a confounder might have been the fact that HRT increases the risk for BC [17, 18]. Among current users of HRT, adjusted RR = 1.66; 95% CI 1.58–1.75, *p* < 0.0001 [18]. However, past users of HRT were no longer exposed to this risk [17, 18]. As some of the study cohorts used LNG-IUS in addition to HRT, it could very likely have influenced the results. Moreover, LNG-IUS is therapeutically used against abnormal bleeding and menorrhagia, which is more common in obese women [19] who are also at higher risk for BC [20]. Another confounder could have been a selection bias because LNG-IUS is more often prescribed to women with a family history of BC [12].

Apparently, the prevalence of BC risk factors in LNG-IUS users is higher than in non-users. For example, they are more likely to have used previous hormonal contraception [9] and have a higher socioeconomic status [12, 14].

Not only does the risk of BC seem to be increased, but other types of cancer also appear to be influenced by LNG-IUS: with 188 more observed than expected cases, the overall cancer risk was elevated [13]. On a positive note, LNG-IUS seemed to significantly decrease other hormonal cancer risks. Two studies found a reduced risk for ovarian and endometrial cancer [9, 13]. This finding was supported by previous studies, where the LNG-IUS seemed to have an antiproliferative and protective effect on the endometrium [5, 21]. On the other hand, there was insufficient evidence that LNG-IUS decreases the occurrence of ovarian cancer [22].

However, the individual results pointed towards an increased BC risk in LNG-IUS users, especially in postmenopausal women and with longer duration of use. This should particularly be considered in patients with a family history of BC or other risk factors present like obesity and higher age. Nonetheless, positive effects of the LNG-IUS such as a reduced risk for endometrial (and possibly ovarian) cancer and the high effectiveness as a contraceptive method should also be put into the balance and weighed against the probable increase in BC risk.

Moreover, other hormonal contraceptive methods, e.g. combined oral contraceptives (COC) seemed to have an increased BC risk too: in women who were currently using COC, RR of a BC diagnosis = 1.24 (95% CI 1.15–1.33, *p* < 0.00001) [23]. Consequently, the effects of LNG-IUS should be put into perspective and compared with other forms of contraception.

## Conclusion

The results of our review show an increased BC risk in LNG-IUS users. The risk increase was especially marked in postmenopausal women and with longer duration of use. However, the findings must be considered with caution as included studies were very heterogenous and possibly influenced by many confounding factors. Nevertheless, the individual risk profile of patients should be taken in account for counselling. According to our results hormone-free alternatives may be discussed in women at high risk for BC.

Furthermore, the positive effects as risk reduction for other hormonal cancers, therapeutical and contraceptive benefits should also be included in the shared decision-making process.

Further well-designed studies with a focus on certain demographic and epidemiologic group are needed to explore the association between LNG-IUS and BC risk.
